# Selection and targeting of EpCAM protein by ssDNA aptamer

**DOI:** 10.1371/journal.pone.0189558

**Published:** 2017-12-15

**Authors:** Walhan Alshaer, Nida Ababneh, Mamon Hatmal, Heba Izmirli, Moujab Choukeife, Alaa Shraim, Nour Sharar, Aya Abu-Shiekah, Fadwa Odeh, Abeer Al Bawab, Abdalla Awidi, Said Ismail

**Affiliations:** 1 Cell Therapy Center, The University of Jordan, Amman, Jordan; 2 Molecular Biology Research Laboratory, Faculty of Medicine, University of Jordan, Amma, Jordan; 3 Department of Medical Laboratory Sciences, Faculty of Allied Health Sciences, The Hashemite University, Zarqa, Jordan; 4 Department of Chemistry, School of Science, The University of Jordan, Amman, Jordan; 5 HMCSR, The University of Jordan, Amman, Jordan; Consiglio Nazionale delle Ricerche, ITALY

## Abstract

Aptamers are molecules that reveal highly complex and refined molecular recognition properties. These molecules are capable of binding with high affinity and selectivity to targets, ranging from small molecules to whole living cells. Several aptamers have been selected for targeting cellular proteins and they have also used in developing therapeutics and diagnostic strategies. Epithelial cell adhesion molecule (EpCAM) is considered as a cancer stem cell (CSC) biomarker and one of the most promising targets for aptamer selection against CSCs. In this study, we have developed a ssDNA aptamer with high affinity and selectivity of targeting the EpCAM protein extracellular domain. The SELEX technique was applied and the resulted sequences were tested on EpCAM-positive human gastric cancer cell line, KATO III, and the EpCAM-negative mouse embryonic fibroblast, NIH/3T3 cells. Ep1 aptamer was successfully isolated and showed selective binding on EpCAM-positive KATO III cells when compared to EpCAM-negative NIH/3T3 cells, as observed by the flow cytometry and the confocal imaging results. Additionally, the binding of Ep1 to EpCAM protein was assessed using mobility shifting assay and aptamers-protein docking. Furthermore, the binding affinity of Ep1 was measured against EpCAM protein using EpCAM-immobilized on magnetic beads and showed apparent affinity of 118 nM. The results of this study could suggest that Ep1 aptamer can bind specifically to the cellular EpCAM protein, making it an attractive ligand for targeted drug delivery and as an imaging agent for the identification of cancer cells.

## Introduction

Increasing evidences over the last decade revealed that a specific population of cancer cells, named as cancer stem cells (CSCs), plays a key role in inducing tumor relapse, metastasis, and resistance to anticancer treatments [1, 2]. Isolation and characterization of these self-renewable distinct clones allow the identification of their specific cell surface biomarkers. Therefore, the discovery of CSCs and their biomarkers could help in developing selective and effective cancer therapeutics through the specific targeting of the successfully identified biomarkers [3, 4].

The CD44, CD133, and EpCAM receptors are the most common surface markers used to identify CSCs [5]. Epithelial Cell Adhesion Molecule (EpCAM; CD326), is a type I transmembrane glycoprotein with an apparent molecular weight of 33–40 kDa. EpCAM is widely distributed in human epithelial tissues and it plays a significant role in cell-cell and cell-matrix adhesion, proliferation and differentiation [6]. EpCAM represents an important cell-surface marker, overexpressed in a variety of human carcinomas including most human adeinocarcinomas and squamous cell carcinomas [7]. For instance, in human ovarian and breast cancers, EpCAM showed significant overexpression levels when compared to normal epithelial tissues. Additionally, EpCAM has been identified as one of the CSC markers, and acted by inducing maintenance of their self renewal and the pluripotent phenotype rather than by antagonizing apoptosis [6, 8–11]. Currently, several trials have been conducted to target EpCAM-expressing tumor cells using specific antibodies [12]. However, the high affinity antibodies are poorly tolerated and induce pancreatitis, while the lower affinity antibodies were better tolerated in patients. Therefore, targeting ligands such as aptamers may represent a promising alternative for antibodies.

Aptamers are emerging as a new class of molecules that can be used in a wide variety of applications including diagnostics, purification processes, drug discovery, and therapeutics [13]. Aptamers are defined as short chemically synthesized single-stranded oligonucleotides of deoxyribonucleic acid (DNA) or ribonucleic acid (RNA) sequences that fold into unique 3-D structures which allow them to interact and bind to their target molecules with high affinity and selectivity [14, 15]. In addition, aptamers show several attractive properties that make them superior to their counterpart monoclonal antibodies. For instance, they are low molecular weight molecules (8–25 kDa), which allows them to penetrate rapidly and reach their target sites *in vivo* [16]. They are mostly non-immunogenic *in vivo*, chemically and thermally stable, and more importantly their synthesis and modification is inexpensive and scalable [17, 18]. The aptamer selection process has been developed using Systematic Evolution of Ligands by EXponential enrichment technique (SELEX), which is a progressive-repeated procedure that aims to select high affinity aptamer sequences from a large combinatorial nucleic acid library that can bind specifically to target molecules [19]. This process involves binding of random sequences to the target of interest, then separation of the bound sequences and amplification of the recovered aptamers by the polymerase chain reaction (PCR) [15, 20]. Aptamers have been studied as bio- probes in diverse diagnostic and therapeutic applications as well as in the production of new drugs and improving drug delivery systems [21]. Additionally, several attempts have been carried out to isolate aptamers against targets involved in various diseases, such as cancer and viral infections [13, 17].

In this study, the *in vitro* selection method, SELEX, was used to isolate ssDNA aptamers against EpCAM protein. Among the resulted aptamers, one aptamer named as Ep1 aptamer, the binding affinity was investigated. Moreover, binding was investigated on human gastric cancers cell line (KATO III) using flow cytometry and confocal imaging. With the assertion of the therapeutic properties of aptamers to work in a way similar to monoclonal antibodies and the development of technologies to efficiently target EpCAM-expressing tumors, the selected Ep1 aptamer represents a promising molecule in diagnostics and developing targeted drug delivery system on EpCAM-expressing tumor cells including CSCs.

## Materials and methods

### Materials

A 90-mer ssDNA template: 5'-GGGATGGATCCAAGCTTACTGG(45N)GGGAAGCTTCGAT AGGAATTCGG-3') was chemically synthesized using cyanoethyl phosphoramidite chemistry with 45- random nucleotides in the central region. The PCR primers for template amplification were 5'-GGGATGGATCCAAGCTTACTGG-3'forward primer, and 5'-CCGAATTCCTATCG AAGCTTCCC-3' reverse primer. Nonspecific sequence (NS): 5'-CCCTACCTAGGTTCGAAT GACCGAGTAACCCACCACCGCAGACCTGCCCAACATACGCTATCCTTAAGCC-3'. The EpCAM recombinant protein was obtained from Abnova (Taiwan). The anti-EpCAM PE-labeled antibodies were from Abcam (USA). Finally, the GST-Taged® protein purification system and the QuantiFluor^®^ ssDNA system was obtained from Promega (USA).

### Cell lines

Human gastric carcinoma cell line KATO III mouse embryo fibroblast cell line NIH/3T3 obtained from the American Type Culture Collection (ATCC) was maintained in Iscove's Modified Dulbecco's Medium, supplemented with 20% heat inactivated fetal bovine serum (Lonza, Switzerland), 100 IU/ml penicillin and streptomycin (Invitrogen, USA) and mouse embryo fibroblast cell line NIH/3T3 was maintained in Dulbecco's Modified Eagle Medium (Lonza, Switzerland), supplemented with 10% heat inactivated fetal bovine serum (Invitrogen, USA), 100 IU/ml penicillin and streptomycin (Lonza, Switzerland). All cultures were kept at 37°C in 5% CO_2_ atmosphere.

### Library synthesis and in vitro selection

**Fig 1:**
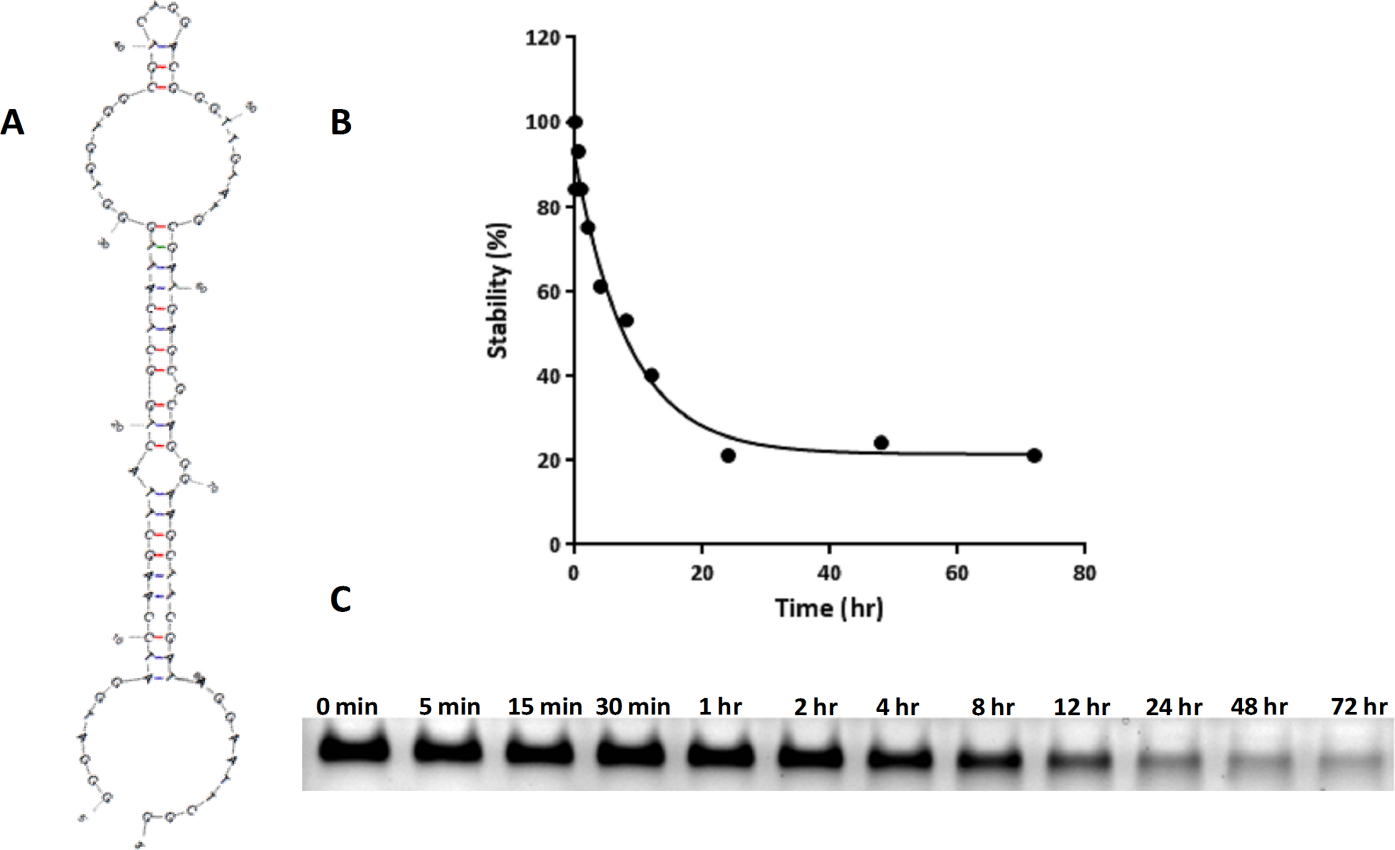
The secondary structure and stability profile of Ep1 aptamer in human serum. (A) Ep1 secondary structure predicted by Mfold. (B) Exponential one phase decay showing the half -life of Ep1 in fresh human serum, (C) Gel electrophoresis shows the stability of Ep1 aptamer in fresh human serum over 72 hr of incubation.

ssDNA library was generated by amplifying the 90-mer ssDNA template by PCR to produce dsDNA library. The PCR mix contained 0.35 nmol of ssDNA library, 5 x Gotaq green buffers, 200 µM of each dNTPs mix, 1 µM of each primer, 2.5 mM MgCl2, and 2.5 U of Taq DNA polymerase (Promega, USA). The PCR conditions were as the following: 5 min at 95°C, and six cycles of short denaturation step for 15 sec at 95°C; then 20 sec annealing at 55°C followed by an extension time of 20 sec at 72°C and a final elongation step of 5 min at 72°C. The asymmetric PCR was applied using different primer ratios, to produce ssDNA library from dsDNA. To perform asymmetric PCR, 20% of dsDNA PCR product was used in 5000 μl of total volume PCR reaction in PCR mix containing 10 mM KCl, 10 mM (NH4)2SO4, 20 mM Tris HCl (pH 8.75), 0.1% Triton-x 100, 0.1 mg/ml BSA, 200 µM of each dNTPs mix (Bio basic inc, Canada), 2 mM MgSO4, 200 U of Taq DNA polymerase (Bio basic inc, Canada), and forward to reverse primer ratio of 50:1. PCR conditions were as the following: 5 min initial denaturation at 95°C, then 25 cycles of 1 min at 95°C, 1 min at 50°C, 1.5 min at 72°C, followed by 10 min final extension at 72°C. After asymmetric PCR, ssDNA pool was separated on 3% low-melting point agarose and the desired ssDNA band was purified by gel extraction. In the first round, a 3.4 nmol of ssDNA library (~2*10^14^ different sequences) was folded by heating at 95°C for 5 min in binding buffer (PBS, 5 mM MgCl2), then rapidly cooled on ice for 5 min, and then equilibrated at room temperature for 10 min. The The ssDNA library was then incubated with 0.025 nmol of GST-tagged EpCAM recombinant protein (Abnova Corporation) for 1 hr at 37°C, followed by separation of unbounded sequences by washing 3 times with 500 µl of MagneGST binding buffer. In the remaining rounds of SELEX, 8 pmol of EpCAM protein was incubated with 34 pmol of ssDNA sequences amplified from previous SELEX round. To increase stringency, number of washing steps has been increased after the first SELEX round into 5 times with MagneGST binding buffer. For negative selection, the ssDNA library was incubated with MagneGST beads for the first five rounds of SELEX to remove nonspecific bound aptamers to MagneGST beads. For each SELEX round, the selected sequences were eluted from the target, and then amplified by PCR and asymmetric PCR. After 11 iterative rounds of SELEX, the resultant aptamers were cloned using pGEM-T Easy vector (Promega) and 17 of the isolated clones were subsequently sequenced by Macrogen Inc. The secondary structure of the selected aptamer was predicted using the Mfold program (Fig 1A).

### Protein binding assay

The binding affinity constant (K_d_) of Ep1 aptamer was determined by incubating different concentrations of the folded Ep1 aptamer (10 to 400 nM) with a fixed concentration of EpCAM protein (10 nM) for 1 hr at 37°C in 20 µl binding buffer. After incubation, MagneGST magnetic particles were added and incubated for 20 min at room temperature and washed three times with 300 µl of binding buffer to remove unbound Ep1 aptamer. The Ep1-EpCAM complexes were then eluted from the beads using 100 µl elution buffer and incubated for 30 min. The fraction of bound Ep1 aptamer was quantified using QuantiFluor® ssDNA System by a GloMax®-Multi Detection System (Promega, USA). The same quantities of Ep1 aptamer was incubated with the GST beads but without EpCAM protein and used as a negative control to determine the nonspecific binding background. The binding data were analyzed to get a saturating curve by performing a regression analysis fitting model for one binding site. Electrophoresis gel mobility shifting assay has been performed by incubating different concentrations of EpCAM protein with fixed concentration of Ep1 aptamer for 1 hr at 37 ^o^C. After that, aptamer-target complexes were running on 9% polyacrylamide gel and stained with SYBR green staining solution for 15 min with constant agitation. The results were examined by Gel documentation system (Bio-Rad). The specific binding was fitted using nonlinear regression analysis for one binding site using the equation: *Y* = (*Bmax* * *X*)/(*Kd* + *X*) While Y is the specific binding, Bmax is the maximum specific binding, X is the Ep1 concentration, Kd is the dissociation constant.

### In vitro stability of Ep1 aptamers

To investigate the stability of Ep1 aptamer against nucleases, 24 µg of Ep1 aptamer was folded in 24 µl binding buffer and then incubated in 100 µl of fresh human serum at 37°C. After that, aliquots of 10 µl were taken at different time points for a total of 72 hr. the aliqutes were treated with 0.6 mAU of proteinase K (Qiagen) followed by incubation at 56°C. The stability of the Ep1 was analyzed on 4% agarose gel. Moreover, the bands intensity has been quantified using Image Lab™ Software (bio-rad) and analyzed by exponential one phase decay.

### Flow cytometry

A total of 1x10^5^ KATO III cells were seeded into 24 well plate and incubated overnight in CO_2_ incubator until they reached 80% confluency. Then, the media was aspirated, and the cells were detached using 0.25% trypsine-EDTA, centrifuged and resuspended in 200 µl of binding buffer. After that, Cy5.5-labeled Ep1 aptamer was added at 200 nM final concentration and incubated at 37°C for 60 min. After incubation, the cells were washed two times using 1 ml of binding buffer and resuspended in 300 μl binding buffer. After that, 10,000 events were counted by BD FACSCANTO II and analyzed using BD FACSDiva™ software version 7.0.

### Confocal microscopy

A total of 2*10^5^ KATO III cells were seeded on cover slips in 12 well plate and incubated overnight for attachment. After that, the cells were washed three times with binding buffer and then incubated with 200 nM of Ep1 aptamer for 45 min at 4°C. Then, the cells were washed again for another three times with binding buffer and then fixed with 4% paraformaldehyde for 10 min at room temperature followed by washing three times with PBS. Cells were then transferred into glass slides and dibbed in mounting medium containing DAPI. LSM (laser scanning microscopy) images were captured with a confocal microscope (LSM 780, Zeiss) using a Plan Apochromat 20x/0.8 M27 objective, ZEN 2012 SP1 software (black edition, Zeiss) and the following settings: frame size 512 x 512; Pinhole Size 30 µm; laser 633nm; Master gain 780.

### 3-D structure prediction of ssDNA aptamers

MC-fold web server, (http://www.major.iric.ca/MC-Fold/) was used for prediction of Ep1 aptamer secondary folding structure and hybridization. Aptamer structure with the minimum energy (the lowest ΔG value) was selected. The Vienna output format (dot-bracket notation) was utilized for construction of the aptamer 3-D structure. Then RNA Composer was then used as a freely available tool over the web (http://rnacomposer.ibch.poznan.pl/Home) for entirely automated prediction of the aptamer 3-D structures. RNA Composer server performs according to the principle of translation machine and executes on the RNA FRABASE database. RNA FRABASE is a search engine associated with database of RNA 3-D structures, which depends as an input on secondary structure in the dot-bracket notation (Vienna format). Secondary structure of the aptamer in the Vienna format obtained from MC-fold analysis was imported into the RNA Composer server and aptamer 3-D structure outcomes were downloaded as PDB file. RNA Composer output is 3-D structure of RNA form of aptamers in which an additional hydroxyl (OH) group is present in 2'-carbon atom of ribose and also thymine was replaced by uracil. The Discovery Studio Visualizer software (windows version 4.1) was used to do these modifications using “Build and Edit Nucleic Acid” tool. Finally, the modified aptamer structures were optimized by 30,000 steps of gradient energy minimization method using NAMD software.

### Docking simulation of Aptamer/EpCAM

The docking simulation was performed between the predicted aptamer and EpCAM using ZDOCK online server (http://zdock.umassmed.edu). ZDOCK as an automated tool searches all possible binding modes in the translational and rotational space between the aptamer and EpCAM and evaluated each pose using an energy-based scoring function. Finally, the best pose was selected for further interaction investigation.

## Results and discussion

### In vitro selection of ssDNA aptamers to EpCAM protein

In human carcinomas, higher expression of EpCAM has been reported in metastases, malignant effusions, and cancer stem cells [11, 22]. In many tumors, the overexpression of EpCAM has been linked to overall all poor prognosis [23]. Down regulation of EpCAM by siRNA showed a decrease in migration and proliferation of human breast cancer cells [24]. Therefore, EpCAM is a potent target for developing antitumor therapeutic and diagnostic strategies. Therapeutic antibodies against EpCAM have been already developed. Such as catumaxomaib (Removab), which was FDA approved for the treatment of malignant ascites in patient with EpCAM+ carcinomas [25]. Although, antibodies hold advantages in their uses as therapeutic molecules, they show high immunogenicity potential and high cost of production [17]. Aptamers represent attractive molecules narrowly close to antibodies in their specificity and target recognition [26, 27]. They have superior properties to antibodies, such as the ease of synthesis, little or no toxicity level production, low or no level of immunogenicity, and they are smaller in size compared to antibodies, which facilitates their use in many biomedical and therapeutic applications including diagnostics, and targeted drug delivery [26].

Recombinant human EpCAM protein was used as a target for *in vitro* selection of ssDNA aptamer sequences. DNA aptamers are less expensive, they possess high shelf life, and they are more stable in biological fluids compared to RNA aptamers [18]. To prepare initial library, a 90nt length ssDNA template composed of 45-nucleotides random region was designed and synthesized. Asymmetric PCR was applied to generate ssDNA library. A pool of 2*10^14^ of ssDNA molecules was folded in binding buffer and incubated with GST-tagged EpCAM protein. After that, the unbound sequences were washed and the aptamers-EpCAM complexes were immobilized on GST-magnetic beads and then amplified by PCR. A negative selection was performed in the first five rounds of SELEX to isolate aptamers that bind to GST-tagged beads thereby eliminating nonspecific sequences. After 11 rounds of SELEX, the final pool were cloned, sequenced and analysed. Among the selected aptamer sequences, one aptamer named as Ep1 showed positive staining on EpCAM expressing tumor cells (S1 Fig). Therefore, Ep1 aptamer was chosen for further binding analysis.

Aptamer stability is important for their effective use in therapeutic or diagnostic applications. They must resist the degradation by exo- and endonucleases. Ep1 aptamer was incubated with fresh human serum for 72 hr, to investigate its stability in *vitro*. The results showed that Ep1 aptamer was stable during the incubation period as observed on the agarose gel electrophoresis (Fig 1B and 1C), analysis of Ep1 bands intensities using exponential one phase decay show half-life of 5.9 hr which may suggest a reasonable stability that makes Ep1 a suitable ligand for diagnostics and therapeutic applications.

### Binding of Ep1 aptamer to EpCAM protein

**Fig 2:**
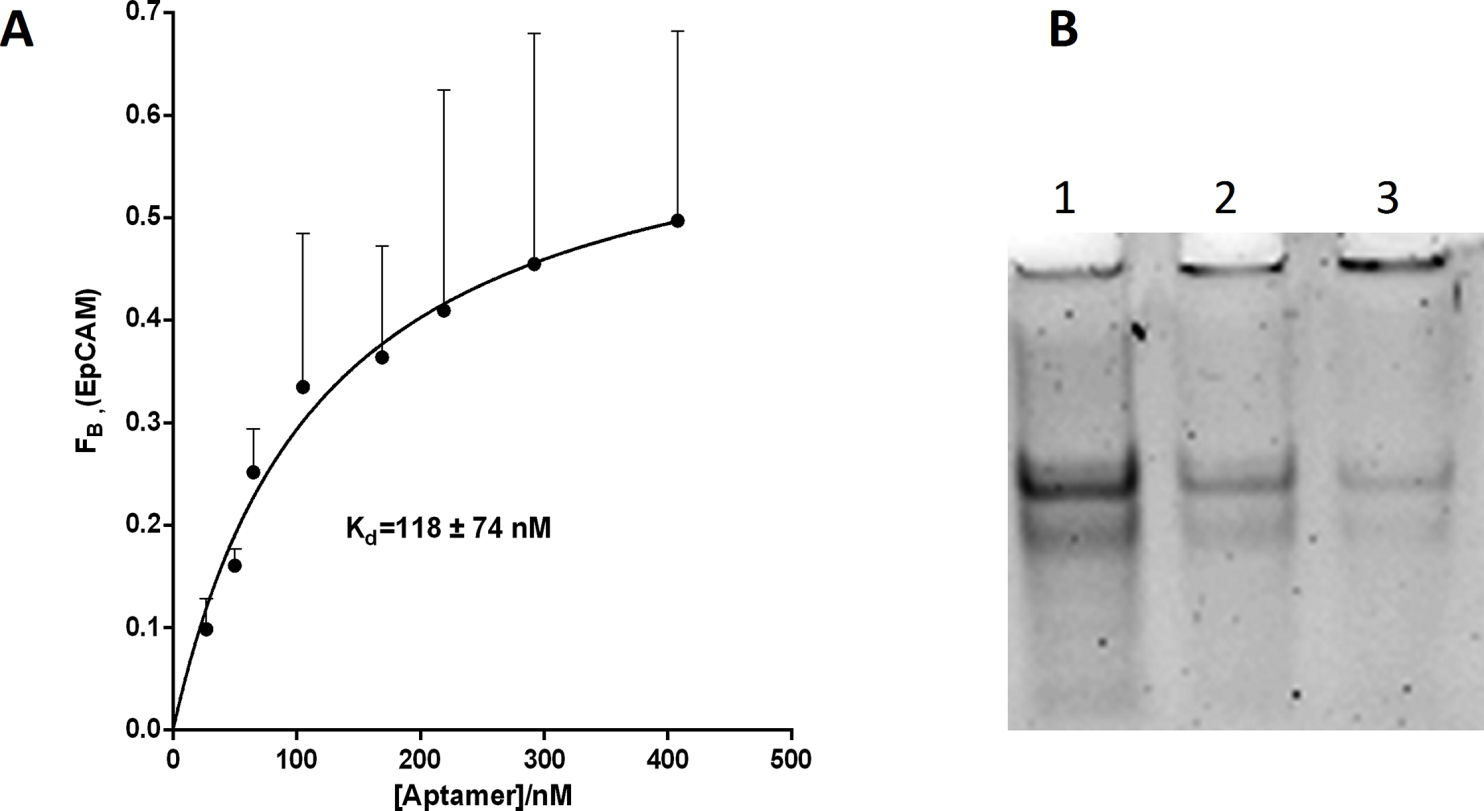
Ep1 aptamer binding to EpCAM protein. (A) Fitting curve analysis to measure the affinity of Ep1 binding to EpCAM protein. The data were fit with regression analysis on one binding site (F_B_ ± SD, n = 3). (B) Gel mobility shifting using different EpCAM protein complexes, the molar ratio of EpCAM:Ep1 were; lane 1, 1:1; lane 2, 2.5:1; lane 3, 5:1.

To test the binding affinity of Ep1 aptamer against EpCAM protein, variable concentrations of Ep1 aptamer were incubated with fixed concentration of EpCAM protein. Negative sequence with the same length was used to exclude background fluorescence. The bound Ep1 aptamer sequences were quantified using quantifluoro ssDNA quantification assay. The bound fraction of EpCAM was also plotted using nonlinear regression of one binding site hyperbola. The results clearly demonstrate the ability of Ep1 aptamer to bind to EpCAM protein with high affinity (K*d* = 118 nM) as shown in Fig 2A. The binding affinity of Ep1 aptamer is in agreement with a previously reported aptamer against EpCAM (55–211 nM) [28], and other CSC markers such as as CD44 (21–83 nM) [29, 30], CD133 (33–145 nM)[31], PSMA (2–11 nM)[32]. For further binding analysis, Ep1 aptamer was incubated with EpCAM protein using different molar ratios of EpCAM protein to Ep1 aptamer. The results showed shifting of the Ep1-EpCAM complex, whereas the free Ep1 aptamer has decreased with increasing EpCAM protein as shown in Fig 2B.

### Binding of Ep1 aptamer to EpCAM-expressing cancer cell line

**Fig 3:**
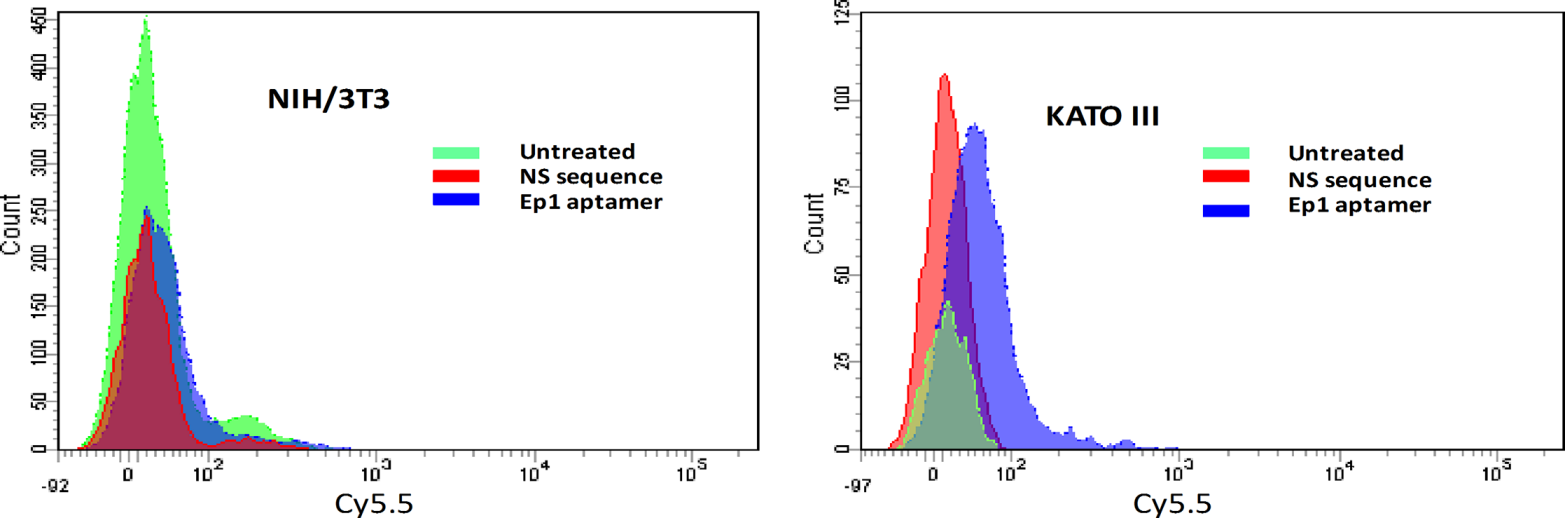
FAC scan histogram analysis of the Ep1 aptamer binding to selected cell lines NIH/3T3 and KATO III. The binding of Ep1 aptamer compared to unstained cells and nonspecific ssDNA sequence.

To investigate the binding selectivity of Ep1 aptamer to EpCAM expressing cells, two cell lines were selected to evaluate the capability and specificity of Ep1 aptamer binding to cancer cells. For positive cellular binding, KATO III human gastric carcinoma cell line was used, which is known for the high level of EpCAM protein expression. Mouse embryonic fibroblast cell line (NIH/3T3) was used as a negative cellular binding control with low EpCAM expression level (S2 Fig). The EpCAM level of expression was examined by flow cytometry and immunofluorescence assays, to investigate the interaction of Ep1 aptamer with the two selected cell lines. The selective binding was demonstrated in the histogram analysis (Fig 3). A Cy5.5-labeled nonspecific sequence with 90 bases length was used as a negative control sequence. Higher binding was obtained for the Ep1 and KATO III cell line combination when compared to NIH/3T3 cell line binding. Moreover, no fluorescent signal was noticed when both cell lines treated with nonspecific sequence (Fig 3).

**Fig 4:**
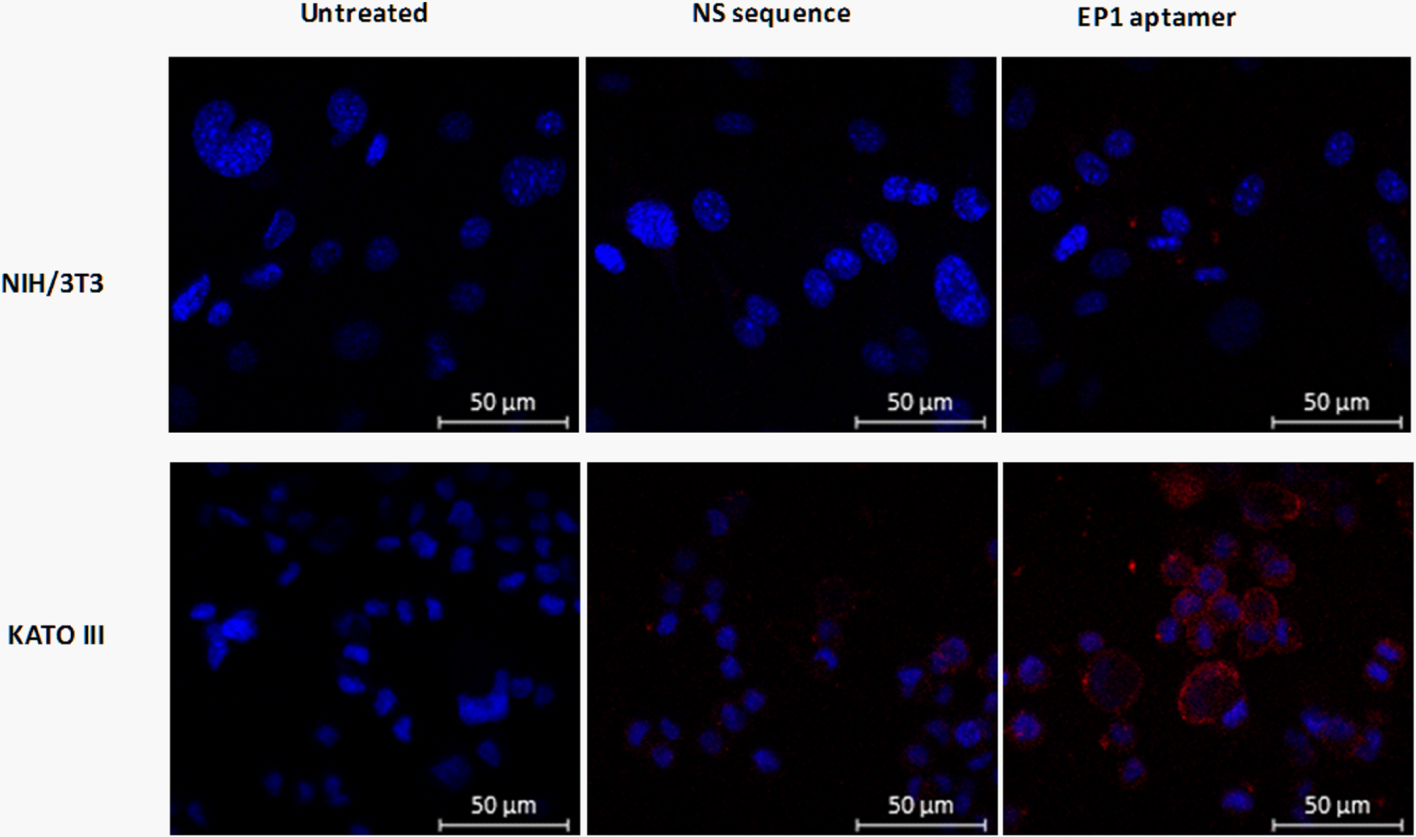
Confocal imaging of KATOIII and NIH/3T3 cells stained with Cy5.5-labeled Ep1 aptamer. The binding of Ep1 aptamer compared to unstained cells and nonspecific ssDNA. DAPI was used as nuclear DNA counter stain.

For further confirmation of the specific binding of Ep1 aptamer to KATO III cells, cells on cover slips were treated with Cy5.5-Ep1 aptamer and imaged by confocal microscopy. The fluorescent intensities showed higher localization of Ep1 aptamer on KATO III cells compared to fluorescent visualized on NIH/3T3 cells or when using the nonspecific sequence (Fig 4).

### Docking of Ep1 aptamer to EpCAM protein

**Fig 5:**
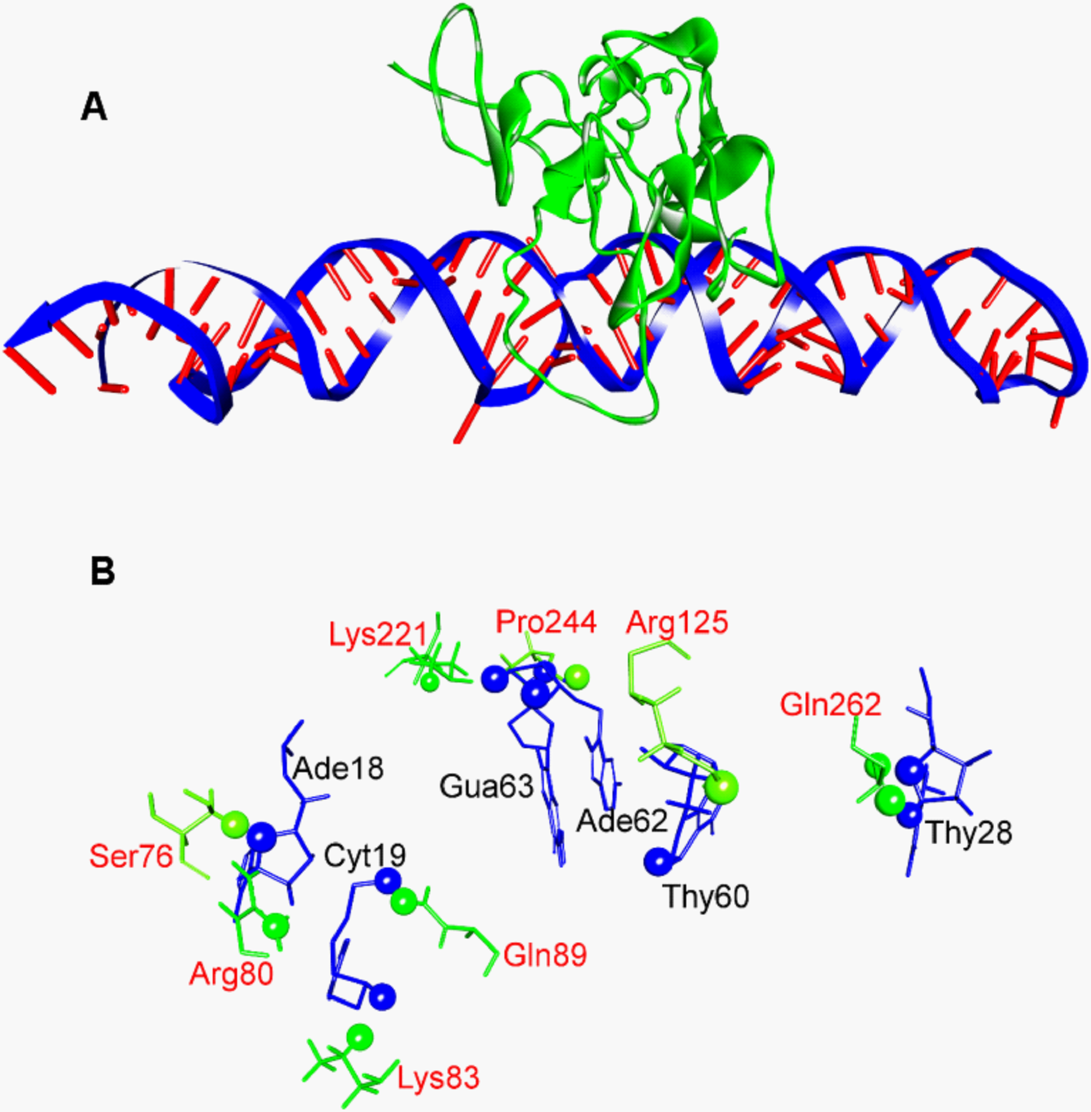
The best-docked pose of EpCAM and aptamer. (A) 3-D representation of the docking. (B) The atoms that are involved in favorable interactions are annotated as spheres.

The best-docked pose is shown in Fig 5A, with the main interacting amino acids are shown in Fig 5B. The fig also shows closely contacting binding atoms (at ≤ 2.5 Ǻ) in the static best docked pose collected from the crystallographic protein complex after docking with the 3-D predicted structure of the aptamer (Fig 5B), the close atoms are annotated as spheres (hydrogen are shown as white spheres). From these contacts, Thy17, Ade18, Cyt19, Cyt23, Thy27, Thy28, Ade59, Thy60, Gua61, Ade62, Gua63, and Ade72 were shown to be the units involved for the protein-aptamer complex interactions. Thy60, Ade62, Gua63, Ade18, Cyt19, and Thy28 being the most important for critical interactions, thus any truncation should avoid these nucleotides. The main favorable interacting atoms (i.e., electrostatic, hydrophobic, and hydrogen bonds) within these residues are ARG80-NH1:CYT19-O2P, ARG125-NH2-THY60:O1P, LYS221-NZ:ADE62-O2P, LYS221:NZ:GUA63:O2P, GLN89-HE22:CYT19:O2P, GLN262-HE22:THY28-O4’,SER76-HB1:ADE18:O4’, LYS83-HE2:CTY19:O3’, GLN262-OE1:THY28-H1’, PRO244-O: GUA63-H5’2.

For DNA, there's no accurate prediction method available, RNA tools are used instead. For example, Heiat et al performed RNA prediction first by RNA Composer server, and then the Discovery Studio Visualizer software was used to modify aptamer 3-D molecular structure from RNA to DNA [33]. Therefore, similar protocols have been applied for docking of Ep1 aptamer to EpCAM protein *in silico*. The docking results are in agreement with the *in vitro* binding experiments. Moreover, in *silico* docking provide a promising tool for prediction of binding nucleotides, thereby aid in decreasing time and efforts needed for *in vitro* experiments to find the minimum size of the aptamer sequence that is capable of binding with high affinity to the selected target.

Finding therapeutics able to target diseased cells is a great challenge in medicine. One way to achieve this goal consists in targeting biomarkers that are specifically or differentially expressed on diseased cells, thereby delivering the drug specifically to these biomarker-expressing cells. Recently, aptamers have been successfully selected to the cancer stem cell biomarkers. Cancer stem cells (CSC) are defined as a small population of cells that are characterized by their ability to renew, proliferate, and differentiate into heterogeneous tumor cells. They are highly resistant against therapeutics and maintain tumor progression. The best described cancer stem cell markers are CD44, CD133, and EpCAM. Therefore, it is not surprising to find several aptamers selected against these markers [30, 34–36].

Although introducing chemical modifications to aptamers during selection process is common to overcome the risk of losing proper folding of the aptamer structure, it is possible to modify aptamers after selection. For instance, the persistent activity of aptamers in physiological conditions can be optimized during- and/or post- selection by conjugation with partners such as polyethylene glycol or cholesterol which can increase circulating half-life. Furthermore, chemical modifications of the aptamers can be incorporated into the sugars or phosphodiester linkages which can enhance nuclease resistance [37].

## Conclusion

In conclusion, the findings of the present study have shown that the selected aptamer (Ep1) binds specifically to EpCAM-expressing tumor cells. This would suggest that the developed EpCAM aptamer could be used in therapeutic applications as a ligand for targeted delivery of cytotoxic drugs into tumors and cancer stem cells, as well as in diagnostic applications for identification of cancer cells.

## Supporting information

S1 Fig Flow cytometry screening for the isolated 17 sequences showing the binding of Ep1 to EpCAM+ KATO III cell line. (TIF)

S2 Fig Flow cytometry analysis for detection of EpCAM expression by control and EpCAM+ cell lines using anti- EpCAM PE-labeled antibodies. (TIF)
