# Correction: Selection and targeting of EpCAM protein by ssDNA aptamer

**DOI:** 10.1371/journal.pone.0194529

**Published:** 2018-03-14

**Authors:** Walhan Alshaer, Nidaa A. Ababneh, Mamon Hatmal, Heba Izmirli, Moujab Choukeife, Alaa Shraim, Nour Sharar, Aya Abu-Shiekah, Fadwa Odeh, Abeer Al Bawab, Abdalla Awidi, Said Ismail

The co-first author’s name is incorrect. The correct name is: Nidaa A. Ababneh. The correct citation is: Alshaer W, Ababneh NA, Hatmal M, Izmirli H, Choukeife M, Shraim A, et al. (2017) Selection and targeting of EpCAM protein by ssDNA aptamer. PLoS ONE 12(12): e0189558. https://doi.org/10.1371/journal.pone.0189558

## References

[1] Alshaer W, Ababneh N, Hatmal M, Izmirli H, Choukeife M, Shraim A, et al (2017) Selection and targeting of EpCAM protein by ssDNA aptamer. PLoS ONE 12(12): e0189558 https://doi.org/10.1371/journal.pone.0189558 29245156 10.1371/journal.pone.0189558 PMC5731996

