# Supplementary figures and images for: Selection and targeting of EpCAM protein by ssDNA aptamer
Source: PLoS One. 2017 Dec 15;12(12):e0189558. doi: 10.1371/journal.pone.0189558 (PMC5731996; doi:10.1371/journal.pone.0189558)

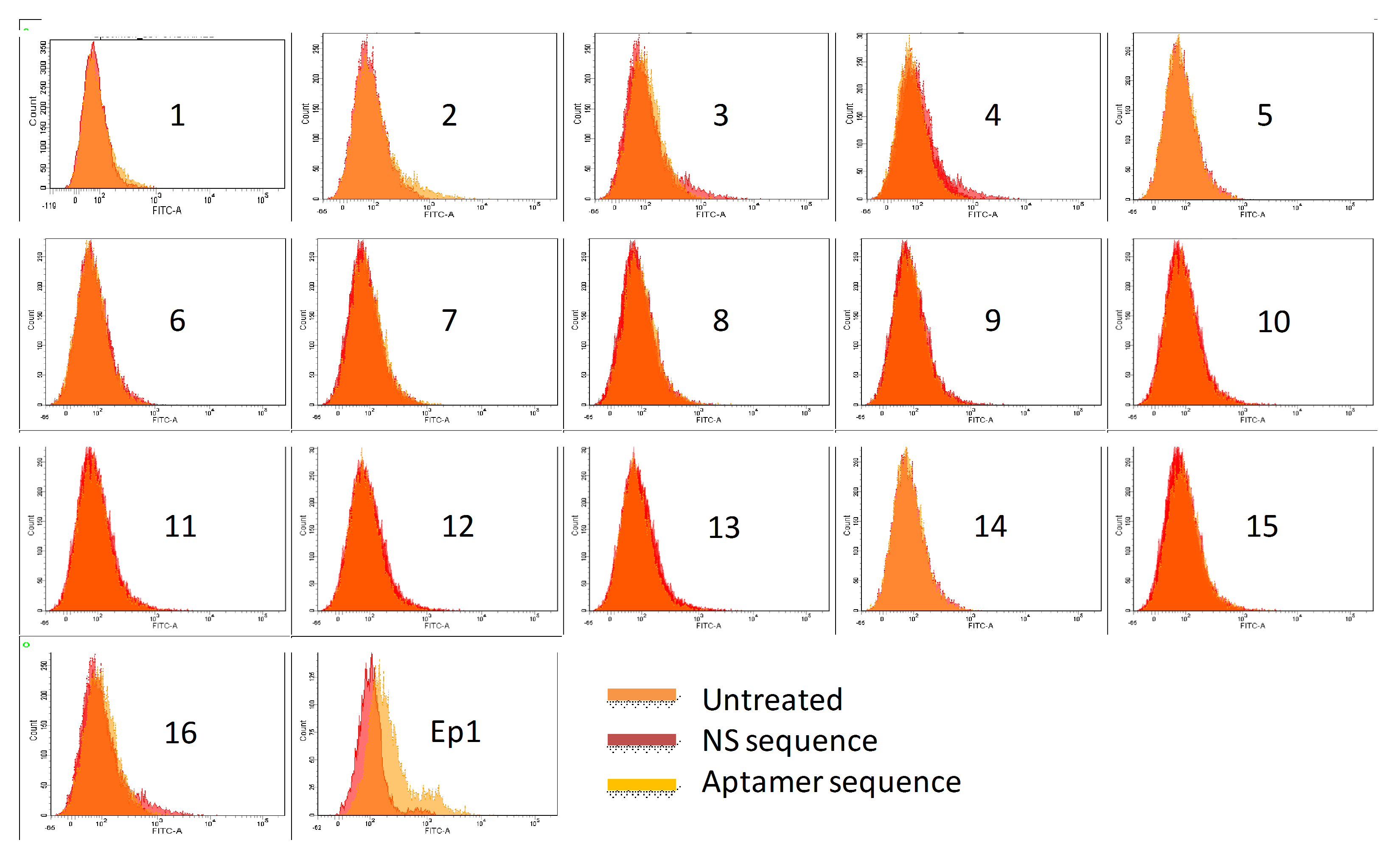

Supplement: S1 Fig — (TIF) [file pone.0189558.s001.tif]

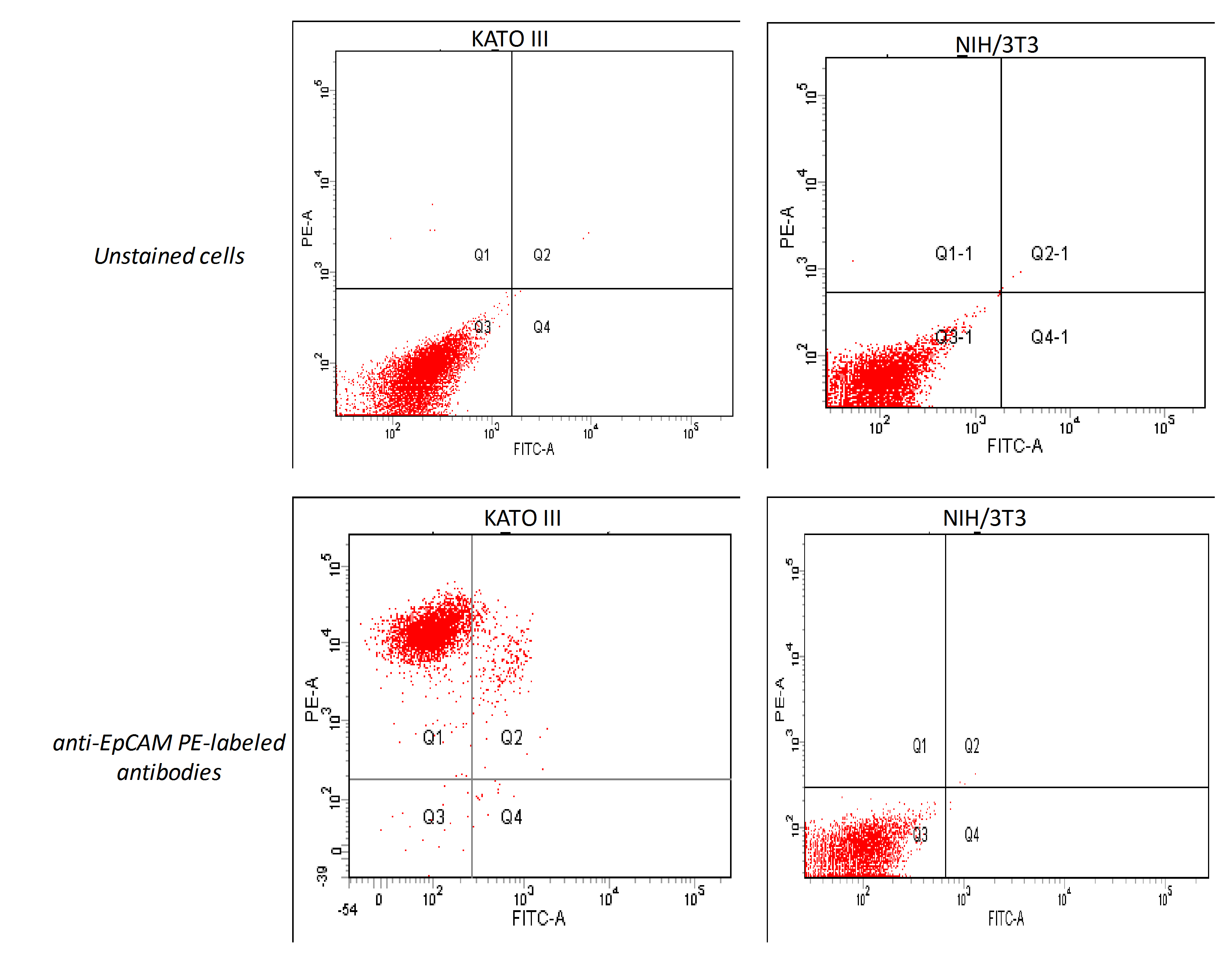

Supplement: S2 Fig — (TIF) [file pone.0189558.s002.tif]
